# Applications of the 1000 Genomes Project resources

**DOI:** 10.1093/bfgp/elw027

**Published:** 2016-07-19

**Authors:** Xiangqun Zheng-Bradley, Paul Flicek

**Keywords:** variation, reference, imputation, GWAS, eQTL, natural selection

## Abstract

The 1000 Genomes Project created a valuable, worldwide reference for human genetic variation. Common uses of the 1000 Genomes dataset include genotype imputation supporting Genome-wide Association Studies, mapping expression Quantitative Trait Loci, filtering non-pathogenic variants from exome, whole genome and cancer genome sequencing projects, and genetic analysis of population structure and molecular evolution. In this article, we will highlight some of the multiple ways that the 1000 Genomes data can be and has been utilized for genetic studies.

## Introduction

The 1000 Genomes Project was launched in 2008 to establish a deep catalogue of human genetic variation that could serve as a baseline for further research into the relationship between genotype and phenotype and for identifying the genetic basis of human disease. At the conclusion of the data generation phase in 2013, the project had amassed 92 terabases of whole genome and whole exome sequences.

Raw data were submitted by the sequencing centers to the sequence read archive as they were generated. Following a coordinated process, data were assessed for quality, aligned to the reference human genome assembly and sequence variants identified [1]. Although raw and processed data were made publicly available as soon as possible, the project conducted and published more comprehensive analyses in three phases characterized by the number of individuals included in the analysis: 180 in the pilot phase, 1092 in phase 1 and 2504 in phase 3 (phase 2 referred to data production only) [2–4]. The final analysis, published in October 2015, incorporates 26 populations from Africa, Asia, Europe and America and contains 88.3 million variants including 84.4 million bi-allelic single nucleotide variants (SNVs), 3.4 million bi-allelic indels and 60 000 structural variants (SVs) consisting of large insertions, deletions, inversions and copy number variants (CNVs). Powered by new variant discovery algorithms, the final release also included ∼475 000 multi-allelic SNVs and indels. In addition, all individuals and their first-degree relatives (when available) were genotyped using high-density microarrays to enable confident phasing and haplotype estimation [4]. The final data set captured > 99% of SNVs with > 1% minor allele frequency (MAF), 95% of SNVs with >0.5% MAF and > 80% indels of MAF > 0.5%. The heterozygous genotype accuracy was 99% for both SNVs and indels. The variants arising from the three consortium publications were deposited into the NCBI database of single nucleotide polymorphisms (dbSNP). As of dbSNP version 141, 61% of the variants in the database were contributed solely by the 1000 Genomes Project and 58% of those not contributed solely by the 1000 Genomes had been validated by the project's results.

While the final 1000 Genomes data set has only recently become available, the data releases that characterized the earlier phases of the project have already given the wider scientific community ample time to use the data described in the pilot and phase 1 publications. Although the three main 1000 Genomes papers have been collectively cited several thousand times, it is surprisingly difficult to catalog complete usage of the data due in part to the complete openness with which the data can be downloaded and redistributed. In the following sections, we will review some of the studies that have leveraged the 1000 Genomes data to illustrate its broad utility.

### Genotype imputation

A major motivation for the 1000 Genomes Project was to provide a dense marker set for the imputation of genotypes in Genome-wide Association Studies (GWAS).

GWAS is a modern and powerful approach for the unbiased mapping of genomic regions associated with specific phenotypes, and it complements other methods of disease gene discovery. Years before the first successful GWAS, the technique of linkage mapping from well-chosen pedigrees successfully identified the causative genes for a number of Mendelian diseases. However, linkage mapping is not feasible for common diseases such as obesity, high blood pressure or diabetes, where multiple loci and variants contribute to disease susceptibility. Mapping complex-trait loci is difficult for a number of reasons, including that the effect size of each risk locus/variant is too small to be detected by linkage mapping, and these characteristics were originally thought to be better suited to candidate gene studies [5]. However, in practice, genes identified by candidate approaches are no longer considered reliable as they often fail to replicate [6]. As an alternative, Risch and Merikangas conceived genome-wide association in 1996 as a method to detect individual causal variants with small effect sizes using population-level linkage disequilibrium [7]. However, the technology necessary to genotype the required hundreds of thousands or millions of markers in thousands or tens of thousands of individuals did not become feasible until a decade later. In 2007 the Welcome Trust Case and Control Consortium reported one of the first successful large-scale GWAS, using arrays with approximately 500 000 markers and the genotypes of 2000 individuals from each of seven common diseases and 3000 shared controls [8]. As of 13 March 2016, according to the NHGRI-EBI GWAS catalog, 2414 GWAS studies have been published and curated with 16 696 unique SNV–trait associations [9].

Most commonly, the location of the significant trait-associated variants in a GWAS study are outside gene coding regions, and those variants with genome-wide significance only account for a small portion of the observed heritability of the trait [10]. These results were initially surprising and have led some critics to argue against the biological relevance of GWAS. However, it is now clear that replicated GWAS regions represent robust biology and are appropriate for comprehensive follow-up studies [11]. That the nature of GWAS results was initially unexpected might have stemmed from the ‘common disease-common variant’ hypothesis that GWAS was based on. The hypothesis states that a common disease arises because the disease-causing variants are of relatively high MAF in the population [7, 12]. Building on this assumption and the idea that genic variants would more likely be functional, the initial genotyping arrays used for GWAS were biased toward finding associations with both common and coding variants.

Today’s GWAS typically assay SNVs using microarrays, and many of the hundreds of thousands or millions of SNVs on these arrays are ‘tag SNVs’ that help to identify known haplotypes. Using a reference panel of haplotypes, imputation computationally fills in other SNVs on the tagged haplotypes. The final individual genotype then has contributions from both the array data and from imputation and is used for genome-wide association that is at higher resolution than would be possible from the array alone and at lower cost than whole genome sequencing. Before the 1000 Genomes data became available, the imputation panel used in most situations was HapMap, a microarray-based variant catalogue and associated haplotype map eventually containing 3.8 million variants with >5% MAF [13]. GWAS using this panel led to some discoveries in disease molecular mechanisms [14, 15], but as the total number of SNVs in the panel is relatively small and these SNVs have the biases toward coding and more common variation described above, the possibility of new insight was limited [8, 16–20].

The release of the 1000 Genomes phase 1 variant catalogue in 2011 inspired a collection of papers describing the fine mapping of genomic loci previously identified by GWAS. These results identified new and previously missed functional variation giving further insight into the molecular basis of coeliac disease [21], prostate cancer [22], glioma [23], type 2 diabetes [24, 25], coronary artery disease [26], epithelial ovarian cancer [27], breast cancer [28], glycaemia and obesity [29] and other diseases. Some studies used population-specific variant panels derived from the 1000 Genomes data to identify differences in disease genetic architecture between populations [24]. The inclusion of a comprehensive collection of indels and SVs in the 1000 Genomes-based imputation panels also helped to identify previously missed, but significantly associated, indels and SVs in existing GWAS [30, 31].

Given the comprehensive nature of the 1000 Genomes phase 3 variant catalogue, using this data set as an imputation panel, instead of either HapMap or earlier 1000 Genomes releases, will significantly increase the number of imputed variants, as demonstrated by an example GWAS for age-related macular degeneration [4]. Imputation accuracy is similar for common variants using either the 1000 Genomes phase 1 or phase 3 data sets, and better results for rare variants are achieved when using the phase 3 data set. However, even with the newest 1000 Genomes data, imputation accuracy for rare variants remains limited especially for non-African populations [4]. Finally, although the 1000 Genomes data are extremely valuable for imputation, they are not appropriate for use directly as a control population in GWAS owing to the variety of populations included.

### Prioritize variants for pathogenicity

With the ever-reducing cost of DNA sequencing, many exome sequencing and cancer genome sequencing projects have set out to find disease-causing novel or rare variants shared among related and unrelated affected individuals. On average, exome sequencing identifies approximately 20 000 SNVs per individual in normal tissue and often more from cancer samples. A majority of these are known and assumed harmless variants that must be filtered to effectively narrow the investigation to rare or novel variants in the search for pathogenic mutations. The 1000 Genomes Project variants, together with those from other public resources such as dbSNP and the National Heart, Lung and Blood Institute Exome Sequencing Project (ESP), have been widely used to establish novelty for variants discovered in re-sequencing projects [32–34]. Assessing pathogenicity of variants by novelty is based partly on the assumption that the ‘known’ variant collections are free of disease-causing variants. However, as the individuals sequenced by the 1000 Genomes Project were not phenotyped at the time of sampling and their health status and medical histories are not known, they may be carriers of pathogenic variants, especially in recessive disease loci [35]. Therefore, a better approach to avoid filtering out true pathogenic variants is to set a MAF cutoff suitable for specific disease and population based on a high-penetrance disease model [35].

An innovative use of the 1000 Genomes data for prioritizing variants is to use the data to categorize genes and genomic regions based on their evolutionary characteristics. Variants from a re-sequencing study can then be sorted into those that reside in genomic regions under strong natural selection and those that do not. The basic idea is that rare variants (MAF  < 0.5%) tend to be recent events and their enrichment can be used to evaluate the strength of purifying selection on a region. The fewer the rare variants in a region, the stronger the purifying selection is for the region. Additional information such as GWAS signals and expression qualitative trait loci (eQTLs) can eventually overlay the evolutionarily significant alleles to prioritize variants that are linked to phenotypes. Evidence of positive selection within the areas under purifying selection can also be signatures for functional importance as variants with MAF that differs drastically among different continental populations are good indications for positive selection [34, 36]. This method of assigning functional importance to genomic areas based on population variant data complements information from multi-species sequence conservation, because regions conserved among mammals can became nonfunctional in recent evolutionary history [37]; this has been used to prioritize variants discovered from cancer ESPs [34]. A genome browser that enables visualization of different levels of natural selection throughout the genome was developed using the 1000 Genomes Project data [38].

More complex and ambitious variant classification methods have also benefited from the 1000 Genomes data. For example, the Genome Wide Annotation of Variants (GWAVA) algorithm integrates many complementary information sources, including functional and evolutionary data, using a modified random forest approach to predict which regulatory variants are more likely to be pathogenic [39]. GWAVA uses several control data sets from the 1000 Genomes Project as part of its training. A separate variant annotation program, the Combined Annotation-Dependent Depletion (CADD) framework, also integrates multiple data types, but using a support vector machine, to distinguish benign from pathogenic variation [40]. CADD uses the 1000 Genomes data to identify recent sequence changes that have become fixed or nearly fixed in the human lineage and then treats these as indicative of benign mutations. This benign set is compared with an equal number of simulated mutations as part of the CADD training.

### Evolutionary genetics and population history

Human genetic variation was largely shaped by the random process of genetic drift and to some degree by population fitness advantages of natural selection, which can include purifying selection to eliminate deleterious variants and positive selection to accumulate advantageous variants [41]. Although natural selection is not the main force behind most of the variation in the human genome, it has been of key interest to population geneticists because of the insight it provides into the history of human molecular evolution and its implications for human genetic models of diseases. Over the past few years, the 1000 Genomes data have been extensively analyzed to address questions of natural selection and population history. Here we will give some examples of such analyses.

A well-understood model of positive selection is the selective sweep, in which a recent advantageous variant is quickly fixated in the population. These events generally result in an unusually long haplotype of low diversity as the nearby neutral variants piggyback with the advantageous variant in the sweep. As reviewed by Fu *et al.*, one example supporting this model is the well-studied lactase persistence alleles, which are prevalent in Europeans but essentially absent from the non-European populations [41]. Another example concerns Tibetans whose adaptations to their high altitude environment are apparently the result of a fast fixation of variants in the EPAS1 locus, a hypoxia-inducible factor previously associated with athletic performance [42], which may regulate tissue oxygenation at high altitudes [43].

Data from the 179 samples of the 1000 Genomes pilot project were used to identify the extent of which classic selective sweeps have been a feature of recent human evolution. This analysis found a decrease in genetic diversity around exons and conserved noncoding sequences, which fits the expectation of a recurrent-sweep model, but, surprisingly, the diversity around human-specific non-synonymous substitutions was not greater than around synonymous substitutions. Moreover, relative to the genome background, amino acid and putative regulatory sites were not significantly enriched in alleles that are highly differentiated between populations. Based on these observations, Hernandez and colleagues concluded that while positive selection is common, fast fixation of a newly occurred beneficial variant by strong selection as suggested by the classic selection sweep model is rare [44]. Other studies using the 1000 Genomes data have investigated different models of positive selection [45, 46].

Although some estimates of the contribution of purifying selection within human regulatory regions have been controversial [37, 47, 48], statistical analysis of the 1000 Genomes data has provided evidence that purifying selection is prevalent and that there is functional constraint in human noncoding sequences [49, 50]. Purifying selection not only affects SNVs: by analyzing the indels found in the 1000 Genomes pilot samples, Montgomery *et al.*, found that indels inside functional sequences are generally exposed to stronger purifying selection than SNVs; and the length of indels is directly correlated with the selection strength [31].

The distribution and sharing of 1000 Genomes variants within and between populations have been modeled to further understanding of human population history and demography. Extensive analyses of the 1000 Genomes data found that African populations carry a larger genetic diversity than non-African populations. While a majority of common variants are shared globally, African populations have the most continental and population-specific variants; in contrast, European and American populations have the least. Statistical modeling of the data essentially confirmed the out-of-Africa theory of human origin and showed a strong and sustained population bottleneck shared among European, Asian and American populations 15 000–20 000 years ago. The bottleneck experienced by the African populations during the same period is much less severe. After the bottlenecks, all but a few populations had recent explosive increases in population sizes [2–4]. Using the 1000 Genomes pilot data, Gravel and colleagues were able to derive demographic parameters for the out-of-Africa model for African, European and Asian descent [51]. Calculations based on the 1000 Genomes Project data from American populations—Colombian, Mexican-American and Puerto Rican—gave good estimates of the genetic contributions of European, African and Native American ancestries to these admixture populations and filled in details of recent human migration history [52]. Analysis of the high coverage data from the 1000 Genomes pilot trios provided evidence for extensive gene flow between Africa and Europe after the divergence of the populations [53].

As demonstrated by the above examples, the 1000 Genomes Project data have facilitated the detection of increasingly subtle signatures of natural selection and enabled statistical tests of different models. The data also offer detailed insight into human demography and population history.

### The impact of genetic variations on gene expression

Using genetic variation data provided by the 1000 Genomes Project has significantly deepened our understanding of transcriptional regulation and its association with diseases. We will review some examples in this section following some background regarding gene regulation.

In the past 10 years, advances in sequencing techniques revolutionized both genome and exome sequencing and the way gene expression is measured. Transcriptome assessment with RNA-seq generates sequence reads from either the entire collection or an enriched fraction of RNA (such as enriched for polyA+) prepared from cells. The reads are aligned back to a reference genome or transcriptome and the read depth at each position is correlated with the abundance of the RNA and the intron/exon structure can be estimated from the coverage of sequence reads [54]. Unlike previous-generation hybridization-based microarray techniques, RNA-seq does not require prior knowledge of gene and transcript sequences and can be of much higher throughput.

With the advances in RNA-seq and the wealth of genetic variation data created by large-scale efforts such as the 1000 Genomes Project, it is increasingly possible to conduct direct investigations into both the genetic basis for gene expression regulation and the impact of genetic variations on traits at the cellular and organism level [55]. Genetic variation responsible for regulating gene expression can often be found by identifying eQTLs, which are genomic regions where sequence variation is correlated with gene expression variation. eQTL mapping leverages statistical methods developed to link continuous phenotypic (i.e. trait) measurements to genotypic data, but is unlike standard QTL mapping in scale. For example, scanning the human genome for eQTLs generally involves millions of comparisons between observed alleles and the measured gene expression phenotypes.

In 2007, large-scale genome-wide eQTL mapping was first reported using genetic markers from the HapMap project and gene expression measurements collected from lymphoblastoid cell lines (LCLs) of the HapMap samples using whole-genome gene expression microarrays [56, 57]. The studies identified SNVs or CNVs that have association signals in *cis* with the expression of a few thousand genes. However, those studies were underpowered for finding association signals in *trans* and the density of the eQTLs was limited. The resolution of such eQTL mapping was enhanced by using RNA-seq to measure gene expression at the transcriptome scale [58, 59].

With the much denser 1000 Genomes genetic variation map, came larger efforts to systematically map eQTLs using RNA-seq data. For example, the GEUVADIS consortium assessed transcriptome-wide mRNA and microRNA (miRNA) levels by RNA-seq in 465 LCLs that were sequenced by the 1000 Genomes Project [60]. The 465 LCLs are from 5 populations and 423 of them were included in the 1000 Genomes phase 1 release (the remaining 42 samples were analyzed later by the 1000 Genomes Project and the GEUVADIS study used imputation based on the 1000 Genomes phase 1 variants). The linkage analysis between the transcriptome variations and genetic variations revealed eQTLs for 3773 genes and eQTLs affecting ratios of alternatively spliced transcripts for 639 genes, creating a comprehensive catalogue of cis-regulatory genetic variants in a single cell type. Although most common effects had been captured by HapMap3 genotype arrays, many of the most significantly associated eQTL variants were novel sites found by the 1000 Genomes Project [61].

In addition to SNVs, the 1000 Genomes resource contains the most comprehensive collection of short indels [4, 31] and larger SVs [62, 63]. eQTL mapping using these data sets suggest that while all types of variants contribute to eQTL identification, indels [31, 60] and CNVs [64] are over-represented compared with SNVs for association with expression variation traits, especially considering the relative counts of indels and CNVs are much smaller than those of SNVs. Further analysis showed that frame-shift indels of length 1, 2, 4, 5 bp were enriched for exon-level gene expression association compared with in-frame indels of length 3 bp, indicating the action of nonsense-mediated decay [31]. In a more recent study, the 1.3 million indels from the 1000 Genomes phase 1 were imputed into three previously published eQTL data sets and used to establish a comprehensive tissue-specific map of indel eQTLs designed for interpreting GWAS hits; the authors anticipate an even better discovery rate using the phase 3 of 1000 Genomes data [65].

Investigations into the biological mechanisms behind gene expression regulation have also benefited from the 1000 Genomes Project data. Genetic variants are known to influence RNA abundance if they alter regulatory elements or change the copy number of genes; they can also influence the relative abundance of alternative spliced RNAs if they interrupt splice sites, stop codons or reading frames. By systematically categorizing regions enriched for or lacking rare genetic variants, the 1000 Genomes data have helped to assess the functional importance of the regulatory elements such as promoters [66] and splice sites [67].

Methods combining 1000 Genomes genetic variants, RNA-seq data and functional regulatory annotation from sources such as the ENCODE project [68] or the Ensembl Regulatory Build [69] can prove powerful in uncovering the molecular mechanisms behind eQTLs. For example, Gaffney and colleagues have found that close to half of all eQTLs they identified occur in open chromatin, and that they are highly enriched in transcription factor binding sites [70]. The eQTLs found in the GEUVADIS project are also highly enriched in noncoding regulatory elements from the Ensembl Regulatory Build, including transcription factor binding sites, DNase1 hypersensitive sites, active promoters and strong enhancers [60]. These kinds of studies both help to explain the biological mechanism of eQTLs and enable the discovery of putative causal variants in GWAS of various diseases.

Another mechanism of gene expression regulation is through noncoding RNAs such as long intergenic noncoding RNA and short RNA molecules such as miRNA. Rich genetic variation data in noncoding RNA and their target genes are useful in investigating the regulatory effect of noncoding RNAs on gene expression. In one example, combining genetic variants from the 1000 Genomes Project and gene expression data collected by four earlier studies, Lu and Clark identified SNVs and indels inside miRNA loci that were strongly associated with the expression level of at least one of the predicted miRNA target genes [71]. They also discovered thousands of variants in the miRNA target genes associated with the abundance of these target genes. Interestingly, the associated SNVs and indels were significantly enriched in the 3ʹUTR compared with introns; the enrichment was even more significant for long indels >100 bp. As miRNA target sites are known to mostly locate at the 3ʹUTR of the target genes, it is likely that the enriched association signals in the 3ʹUTR directly impact on the target recognition of miRNA [71]. More recently, 1000 Genomes data were used to establish comprehensive genetic variation maps of human miRNA [72] and variation maps of miRNA recognition element seed sites [73], allowing efficient investigations of eQTLs in the miRNA genes.

### Other applications

In addition to the above four categories of common applications, the 1000 Genomes data have been used widely for other purposes. For example, the data were used by the Genome Reference Consortium (GRC) to improve the human reference genome at several thousand locations by identifying ‘a number of bases and indels in GRCh37 that were never seen in any individuals, suggesting they may represent errors in the assembly’ (http://genomeref.blogspot.co.uk/2013/12/announcing-grch38.html). The 1000 Genomes data also contributed to the GENCODE pseudogenes resource by highlighting a list of pseudogenes that are potentially under selection [74].

When mapping short sequence reads to a reference genome, alignments around indels are error prone, leading to false SNVs that are called around indels. A functionality known as IndelRealigner included in the GATK toolkit makes use of known indels and improves the alignment around them (https://www.broadinstitute.org/gatk/gatkdocs/org_broadinstitute_gatk_tools_walkers_indels_IndelRealigner.php). The indel data set from Mills *et al.* [75] has been widely used as the known indels for IndelRealigner, and we anticipate that because of its completeness and high validation rate, the 1000 Genomes phase 3 indels will be used for this purpose in the future.

As the 1000 Genomes Project data is readily available and free to use, it has found value as a good test data set for developing computational tools and statistical models in cases that do not require data from trios or related individuals. In one such example, 1000 Genomes data were used as a test set to verify the genomic segmentation model derived from the human-orangutan alignment [76]. In a more recent example, a novel computational protocol was developed to screen for spinal muscular atrophy carriers based solely on individual exome data. The authors used the 1000 Genomes samples as test data and found that the carrier frequencies in multiple populations match with those reported in the current literature [77].

High-throughput genotype arrays such as Affymetrix GeneChips and Illumina BeadChips are capable of assaying 1 million or more SNVs at one time and have played a key role in finding disease associations in various GWAS. The content of these chips has generally been derived from human genetic variation catalogues produced by large international consortia, with earlier versions based on HapMap data and today’s newest chips on the 1000 Genomes data. Different manufacturers have different criteria to select tag SNVs based on their specific requirements of genome coverage, population and cost for performing the assays in hundreds or thousands of samples [78–80].

## Discussion

In the 5 years since the first release of the 1000 Genomes results, the research community has embraced this comprehensive resource of human genetic variation and a flagship example of an open data project. As a result, many studies have been published using the 1000 Genomes Project data. Four main applications of the 1000 Genomes data have dominated the usage thus far. First is imputing un-assayed genotypes for GWAS. The completeness of the catalogue, especially the inclusion of rare variants with MAF as low as 0.1%, significantly improves the resolution for GWAS, leading to discoveries of new and previously missed causal variants with biological significance for many diseases. Second is to aid screening for pathogenic variants in exome sequencing or cancer genome sequencing projects on well-defined disease cohorts. The 1000 Genomes data can be used to filter out common germ line variants that are not pathogenic. Additionally, the 1000 Genomes data can be used to measure the level of purifying selection. Chromosomal regions that are under high purifying selection pressure tend to be functionally important; variants inside these regions are more likely to be causal for certain phenotypic traits. Third, as the 1000 Genomes data are a dense catalogue of human genetic variants with genotype data in many different populations across the globe, it is naturally a good resource for population genetics. Investigations into the signatures of natural selection, adaptation, human origins and population migration history have all used the data effectively. A fourth major application arises by combining 1000 Genomes data with RNA-seq data and functional regulatory annotation data to identify and characterize a comprehensive list of eQTLs and to prioritize GWAS hits by their associations with eQTLs. Many of the projects that have used the 1000 Genomes Project data are themselves characterized by open science principles and a comprehensive survey of whether studies using the 1000 Genomes data are more likely to be open would be interesting.

One may question the future usefulness of the 1000 Genomes data as newer whole genome sequencing projects are carried out or proposed with even more samples. One such project, the UK10K project, used a low-coverage whole-genome sequencing strategy to identify variants from close to 4000 healthy individuals from two well-studied British cohorts; furthermore, they searched for causal mutations for three types of diseases (rare disease, severe obesity and neurodevelopmental disorders) by high-coverage exome sequencing of 6000 patients [81]. The variants discovered from the 4000 healthy individuals have been used as an imputation panel for the GWAS analysis on the 6000 diseased individuals [82]. Another project, funded by the UK government, plans to sequence 100 000 whole genomes from patients registered and treated by the National Health Service by 2017 (http://www.genomicsengland.co.uk/the-100000-genomes-project/). For both the UK10K project and the 100 000 Genomes project, the data sets are derived solely from the British population and, while they may be more useful for imputing missing genotypes in GWAS of British or other European populations, they do not have the global diversity of the 1000 Genomes Project. In addition, these two projects represent genome sequences from individual patients or various disease cohorts and they may not be suitable for all of the applications reviewed here. Finally, unlike the 1000 Genomes Project data, which is completely open to the entire community, essentially all larger, disease-related projects will have various access policies to their data to ensure participant or patient privacy. Thus, we can predict, as a public human genetic variation reference, the 1000 Genomes data will continue to be useful because of the unique combination of its high quality, global diversity, unbiased health status of the project participants and the open data access policy.

The 1000 Genomes Project has finished, but with the support of the 1000 Genomes Project consortium and funding from the Wellcome Trust, the data resource will be maintained and improved. This extension to the 1000 Genomes Project is known as the International Genome Sample Resource (IGSR) and has recently finished re-mapping of all 1000 Genomes sequencing reads to the recently released GRCh38 human reference assembly, and will soon release variant calls native to the new assembly. These updated alignment and variant sets will benefit from the error correction and gap filling in the new assembly. The IGSR will expand the global catalogue of freely available sequence information by incorporating new data generated using the 1000 Genomes model of consent for open data sharing. Expected future data sets include data from Russian samples, additional African populations and whole genome sequences from the Simons Genome Diversity Project. From samples with genome sequences already included in the resources, the IGSR will also collect complementary functional genomics data sets including RNA-seq, ChIP-seq or other epigenomics data sets, as long as there is data for at least two sub-populations of 100 individuals. For example, RNA-seq data for European populations was generated by the GEUVADIS project [60] and will soon be available for African populations. Through these efforts, we believe that the 1000 Genomes Project and the IGSR will continue to serve the science community in many different ways well into the future. To enhance data accessibility, IGSR developed a new data portal, http://www.internationalgenome.org (also available at http://www.1000genomes.org), which lists all populations and samples of the project and also supports the query and download of data files for specific samples.

Key Points

- The 1000 Genomes resource contains a comprehensive collection of genetic variants in the human genome, all phased onto high-quality haplotypes.
- As a dense imputation panel, 1000 Genomes data have been used to discover new and previously missed causal variants in many Genome-wide Association Studies.
- The 1000 Genomes data offers a great insight into human evolution and population history.
- Combining 1000 Genomes data, RNA-seq data and functional annotations of regulatory elements is a powerful way to study gene expression regulation.
- The 1000 Genomes data will be maintained and improved by a new project known as the International Genome Sample Resource.
